# Comparison of Hepatic and Nephric Total Mercury Concentrations Between Feral and Ranch American Mink (*Neovison vison*) from Northwestern Poland

**DOI:** 10.1007/s00128-012-0555-5

**Published:** 2012-02-23

**Authors:** Elzbieta Kalisinska, Halina Budis, Natalia Lanocha, Joanna Podlasinska, Ewa Jedrzejewska, Danuta I. Kosik-Bogacka

**Affiliations:** 1 Department of Biology and Medical Parasitology, Pomeranian Medical University, Powstancow Wielkopolskich 72, 70-111 Szczecin, Poland; 2 Department of Environmental Management and Protection, Western Pomeranian University of Technology, Słowackiego 17, 71-374 Szczecin, Poland; 3 Warta Mouth National Park, Chyrzyno 1, 69-113 Gorzyca, Poland

**Keywords:** American mink, Total mercury, Kidney, Liver, Bioindicator, Poland

## Abstract

For many years the American mink (*Neovison vison*) has been used in North America (where it originates from) as a sensitive indirect bioindicator in assessing the degree of mercury (Hg) contamination in terrestrial ecosystems. The aim of this paper was the determination of total concentrations of Hg in the liver and kidneys of feral and ranch mink from the Warta Mouth National Park (WMNP) and from farms located in northwestern Poland, for comparison with similar data on American mink from North America. In road-killed feral mink from the WMNP, the mean concentrations were 11.8 and 14.1 mg/kg dry weight in the liver and kidney, respectively. Mean Hg concentrations in feral mink were from 240 to 90 times higher in these two respective tissues than in ranch mink. The feral mink from northwestern Poland had concentrations of hepatic and nephric Hg similar to the highest concentrations that have been recorded over the past several decades in wild American mink from certain areas of Canada and the USA.

American mink (*Neovison vison*, formerly *Mustela vision*) in its homeland, North America, has been used for decades as a sensitive indirect bioindicator for the assessment of mercury contamination of terrestrial ecosystems. Its diet consists largely of fish caught in inland waters, and birds and mammals in the vicinity of rivers and lakes (Carmichael and Baker 1989; Evans et al. 2000; Bartoszewicz and Zalewski 2003).

The liver and kidney of aquatic and semiaquatic predatory vertebrates accumulate methylmercury, the most toxic and widespread form of mercury (Hg), which is subject to biomagnification in aquatic food chains. It tends to reach its highest concentrations in piscivorous birds and mammals, including the mink (Wobeser 1976; Osowski et al. 1995; Scheuhammer et al. 2007). This species belongs to a group of fur-bearing mammals, and therefore in different parts of the world (including Europe) is farmed for fur. More than 50 years ago in Europe (including the territories of the former Soviet Union on the eastern Polish border), ranch mink were deliberately released into the wild. Their descendants, as well as those of mink that had escaped from farms, currently live in their respective habitats in many parts of the continent, including Poland (Lariviere 1999; Bartoszewicz and Zalewski 2003; Brzezinski et al. 2010). In western Poland, including Slonsk Reserve (presently part of the Warta Mouth National Park), American mink did not first appear until the early 1990s (Bartoszewicz and Zalewski 2003).

The aim of this study was to examine the concentrations of mercury in the bodies of road-killed feral mink from the Warta Mouth National Park (Park Narodowy Ujscie Warty) and ranch mink from a farm located in northwestern Poland, for the purpose of comparing their liver and kidney levels, and to assess the levels of contamination in these organs relative to levels reported in North American mink.

## Materials and Methods

**Fig. 1:**
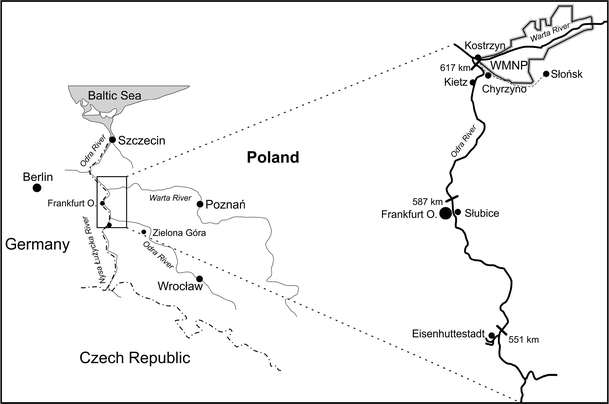
Location of study areas in western Poland

The area of the Warta Mouth National Park (WMNP, 8,074 ha) is periodically inundated by the waters of the Warta and Odra Rivers (Polish/Czech: Odra; German: Oder). The Odra River is the second largest river in Poland and its greatest eastern tributary, the Warta, joins near the town of Kostrzyn (52°35′N; 14°40′S) in western Poland (Fig. 1).

The study was carried out on six feral mink (*N. vison*) found dead on the roads of WMNP in 2009–2011. From these specimens, we collected 6 livers and 8 kidneys for chemical analysis as some organs were damaged. We also examined both the liver and kidneys of 7 ranch mink obtained from one of the farms in northwestern Poland in 2007. Carcasses were kept in a freezer at −20°C. After dissection of the thawed carcasses, samples of the liver and kidney (about 10 g each) used for the determination of Hg were dried at 55°C. Within 4–6 weeks, the samples were weighed three times to constant weight (to 0.1 mg, Sartorius BP221S balance), which made it possible to determine weight-based water content. Subsequently, the samples were crushed in an agate mortar. Total Hg concentrations were determined using atomic absorption spectroscopy (AAS) at the Department of Environmental Management and Protection, Western Pomeranian University of Technology in Szczecin. The assays were run in an AMA 254 mercury analyser (Altach Ltd, Czech Republic) in accordance with previously described methods (Kalisinska et al. 2009). The apparatus has a detection limit of 0.01 ng and a detection range of 0.05–600 ng. The AMA 254 analyzer permits the determination of Hg in biological samples without having to perform prior mineralization in wet conditions. Mercury concentrations were referred to both dry weight (dw) and wet weight (ww) of the respective organ. The analytical procedures used were checked by determining Hg concentrations in samples of two reference materials: bovine liver (BCR 186, Commission of the European Countries, Community Bureau of References—BCR) and fish tissue (IAEA 407, International Atomic Energy Agency—IAEA, Austria).

In addition, the percentage of water content was determined by gravimetric method in the studied organs. Intra-group and inter-group comparisons of mean hepatic and nephric mercury concentrations were performed using a Student *t* test.

## Results and Discussion

The mean water content in the liver and kidney of all studied mink was 69% and 73%, respectively. Our analysis of certified reference materials yielded total Hg concentrations of 1.96 ± 0.16 and 0.237 ± 0.002 mg/kg dw for BCR 186 (n = 6) and IAEA 407 (n = 5), respectively. Corresponding values from the suppliers were 1.97 and 0.222 mg/kg dw, respectively. The recovery rates were 99.2% and 107% for BCR 186 and IAEA 407, respectively.

In feral mink from the WMNP the mean concentrations of Hg were similar in the liver and kidney, the organs that play a key role in the detoxification of Hg—about 12 and 14 mg/kg dw, respectively (Student test *t* = 0.910, *p* > 0.381). In the feral mink, the mean concentration of Hg in the kidney was about 20% higher than in the liver, while statistically confirmed differences between the two types of samples analyzed were not found. Maximum concentrations of hepatic and nephric Hg reached ~21 and ~19 mg/kg dw, which corresponds to 6.5 and 5.1 mg/kg ww, respectively (Table 1). To date, the baseline concentration of Hg in the liver and kidney of the American mink, which would reflect the geochemical background northwestern Poland, has not been determined definitively. Osowski et al. (1995) studied the concentration of mercury in the kidneys of several populations of American mink from Georgia, North Carolina and South Carolina (USA). In the 1960s, a decline in this species was observed. The Piedmont Mountains population had a stable size and was treated as a reference group, known as the Piedmont reference mink (PR). In this PR group, the mean nephric mercury level was 0.527 mg/kg ww (~2.11 mg/kg dw), and this value was taken as reflecting the geochemical background. Carmichael and Baker (1989), on the basis of various papers, suggested “normal” concentrations of mercury in mink liver were ≤2 mg/kg ww (about 6 mg/kg dw).

**Table 1:** Hepatic and nephric mercury concentrations (mg/kg dw and ww) in feral and ranch American mink from Poland (AM—arithmetic mean; SD—standard deviation)

American mink		Liver			Kidney		
		n	dw	ww	n	dw	ww
Feral mink	AM ± SD	6	11.8 ± 6.21	3.65 ± 1.93	8	14.1 ± 3.31	3.81 ± 0.89
	min–max		3.28–20.9	1.02–6.47		9.34–19.0	2.52–5.12
		1^a^	0.11	0.03	1	0.31	0.08
Ranch mink	AM ± SD	7	0.05 ± 0.02	0.01 ± 0.01	7	0.15 ± 0.11	0.04 ± 0.03
	min–max		0.04–0.08	0.00–0.03		0.06–0.37	0.02–0.10

^a^specimen that was probably a recently escaped ranch mink

The main anthropogenic sources of heavy metals (including Hg) in the Odra River course are the Upper and Lower Silesian coal-mining and metallurgical districts. Also important sources of heavy metal pollutions are German cities, Eisenhuttenstadt and Frankfurt O. (Oder), where major industrial enterprises are located, e.g. the steelworks of the Eisenhuttenkombinat-Ost (Muller et al. 2002). Moreover, the contamination of the confluence of the Warta and Odra rivers with mercury may have been caused by a celulose-paper production plant in Kostrzyn (founded in the 1930s). In 2002, the concentration of Hg in sediments of the Odra ranged from 0.35 to 1.32 mg/kg in Eisenhuttestadt and from 0.28 to 1.49 mg/kg in Kostrzyn (Fig. 1) (Boszke et al. 2004). In 2009, results from PGEMO of bottom sediments showed 0.25 mg Hg/kg in the Warta River near Kostrzyn (www.gios.gov.pl). The sediments were classified as weakly and moderately polluted, respectively (Bojakowska and Sokolowska 1998). According to US sediment quality guidelines (SQGs) mercury should not exceed 0.2 mg/kg dw (MacDonald et al. 2000). In relation to the quoted value of SQGs used in ecotoxicological studies, the concentration of Hg in sediments of the Warta River in 2009 was 25% higher, and in earlier years many times higher for both the sediments of the Odra and Warta.

Interesting observations on the difference in hepatic Hg concentrations in mink from various parts of the Great Lakes (Quebec) were made by Martin et al. (2011). Canadian mink, which were trapped in large riverine marshes, had the highest hepatic Hg level (15.3 mg/kg dw) in comparison to individuals collected in smaller tributaries/creeks or drainage ditches of the basin (2.3 mg/kg dw) (Table 2). Our feral mink were found in areas regularly flooded by the Odra and Warta rivers. From this, it appears that feral mink collected from the WMNP had elevated total Hg levels in comparison to the suggested geochemical background level for Hg. Recently, Bowman et al. (2012) drew attention to the differences in mercury concentrations in the liver from wild and feral mink collected in Ontario (Quebec) in 1998–2006. Among 133 genotyped individuals of American mink, the authors found 9% domestic, 10.5% hybrid and 80.5% wild genotypes, respectively. After exclusion of domestic mink from a general pool of specimens, wild mink contained a mean of 3.91 mg/kg dw total Hg in their liver, compared to a mean of 1.33 mg/kg dw in domestic mink. In comparison to the hepatic Hg concentration of wild mink from Canada, Polish feral mink had over three times higher mercury levels (~12 m/kg dw). However, among the feral mink in our study there was a single individual (excluded from statistical analysis), which had distinctly lower concentrations of Hg in both the liver and kidney. These concentrations were much more similar to the values indicated in the organs of Polish ranch mink (Table 1). It seems, therefore, that in an indirect assessment of environmental contamination with mercury, one should exactly identify individual specimens included in the chemical analysis, as recently escaped ranch mink from farms (or domestic mink) may cause underestimations of contamination in the study area.

**Table 2:** Comparison of mean mercury concentrations in the liver and kidney of feral American mink from Poland and wild mink from North America (mg/kg dw and ww; in the dw and ww conversions it was assumed that both the liver and kidney contain 70% water; LFR, Lower Fraser River; WMNP, Warta Mouth National Park)

Liver		Kidney		Country and years	Source
dw	ww	dw	ww		
11.8	3.65	14.1	3.81	Poland, WMNP, 2009–2011	This study
1.33	0.40	–	–	USA, South Carolina, 1987–1988	Carmichael and Baker 1989
–	–	7.47	2.24	USA, North and South Carolina, 1989–1991	Osowski et al. 1995
2.87	0.86	–	–	USA, Main, 2001	Bank et al. 2007
14.57	4.37	7.30	2.19	Canada, Québec, 1993–1995	Fortin et al. 2001
5.10	1.53	3.67	1.01	Canada, Ontario, 1994	Evans et al. 2000
12.6	3.79^a^ (±4.32)	–	–	Canada, Ontario, 1998–2006	Bowman et al. 2012
0.99^a^ (±0.36)–7.31 (±1.52)	0.30	–	–	Canada, Great Lakes, 1998–2006	Martin et al. 2011
3.07	0.92	2.20	0.66	Canada, Yukon, 2001–2003	Gamberg et al. 2005
4.62	1.37	3.13	0.94	Canada, British Columbia, Lower Fraser River, 1990–1996	Harding et al. 1998

^a^mean ± SE

In native wild specimens of the American mink, nephric and hepatic concentrations of Hg have assumed very different values, depending on the region of North America, as well as the degree and source of mercury contamination. Over the last two decades, in North American trapped mink, the mean liver and kidney concentrations, have usually been between <5 and 10 mg/kg dw (Table 2).

Maximum concentrations of Hg in the liver and kidney of our ranch mink did not exceed 0.09 and 0.40 mg/kg dw, respectively. In the ranch mink the mean concentration of Hg in the kidney was almost three times higher than in the liver (*p* < 0.04). Similarly, Wobeser (1976) and Stejskal et al. (1989) either did not detect mercury in the liver and kidney in ranch mink, or its concentrations were low (<0.7 mg/kg ww or <2.3 mg/kg dw).

Comparative analysis showed statistically confirmed differences in hepatic Hg concentrations between escaped ranch mink and feral mink (*p* < 0.03), and escaped ranch mink and ranch mink (*p* < 0.01).

In summary, feral mink from the Warta Mouth National Park, when compared with ranch mink from northwestern Poland, had mean hepatic and nephric total Hg concentrations (dw) that were on average about 240 and 90 times greater than their respective concentrations in ranch mink. Due to the small number of samples tested, it is desirable to continue these studies on larger and more diverse materials to allow better assessment of the situation of degree of Hg concentration in various piscivorous birds and mammals that inhibit the WMNP, including American mink and protected Eurasian otter. This study is one of the first in Europe to describe the use of feral American mink as bioindicators to indirectly assess of mercury pollution in the environment.
